# Investigating health professionals' perspectives and experiences of food security‐related conversations in diabetes care

**DOI:** 10.1111/dme.15470

**Published:** 2024-11-09

**Authors:** Sophie Mohamed, Alison Avenell, Flora Douglas, Andrew Keen

**Affiliations:** ^1^ NHS Grampian, JJR Macleod Centre for Diabetes Endocrinology and Metabolism Foresterhill Aberdeen UK; ^2^ Health Services Research Unit University of Aberdeen Aberdeen UK; ^3^ School of Nursing, Midwifery and Paramedic Practice Robert Gordon University Aberdeen UK

**Keywords:** diabetes care, diabetes self‐management, eating behaviours, food insecurity, poverty, social determinants of health

## Abstract

**Aims:**

Household food insecurity (FI) is a serious public health concern and disproportionately affects people living with chronic health conditions, undermining diabetes self‐management. Little is known about healthcare professionals' (HCPs) experiences of supporting people affected by diabetes and FI, and no national guidelines incorporate consideration of FI within UK diabetes care. A qualitative study of NHS HCPs' consideration of FI within diabetes care, and the extent to which it informs their clinical practice, was undertaken.

**Methods:**

Fifteen HCPs providing self‐management support to people with Type 1 or Type 2 diabetes in a Scottish Health Board took part in semi‐structured interviews. Data were analysed using a thematic framework approach informed by the Capability, Opportunity, Motivation and Behaviour (COM‐B) model of behaviour change.

**Results:**

Although the potential impact of FI on diabetes self‐management was recognised, this important consideration was not currently core to their clinical practice. Enablers and barriers identified included: personal feelings about raising the issue, lack of knowledge of available resources, the patient‐practitioner relationship, and the wider socioeconomic environment. Practical suggestions to support HCPs included: specific training on communication, access to patient support information, use of a screening tool to assess FI, and building NHS‐third sector links.

**Conclusions:**

Our findings provide insight into cognitive factors, emotional processes and environmental systems impacting on HCPs' practice supporting individuals with diabetes and FI. Research with affected patients is needed to gain a better understanding of how to provide support within NHS settings.

**What's new?**

- Little is known about healthcare professionals' experiences of supporting people affected by diabetes and food insecurity, and no national guidelines incorporate consideration of food insecurity within UK diabetes care.
- The consideration of food insecurity is not currently core to routine clinical practice in diabetes. Specific barriers and enablers to healthcare professionals discussing food insecurity with patients and appropriately adjusting care were identified.
- There are practical steps that could be taken to support healthcare professionals and their patients, including staff training, increasing the accessibility of support information, self‐report pro forma, and NHS‐third sector links and pathways.

## 1 INTRODUCTION

Food insecurity (FI) is characterised as ‘the inability to consume an adequate quality or quantity of food in socially acceptable ways or the uncertainty that one will be able to do so’,^1^ and is associated with poverty, mental and physical illness, and social isolation.^2^, ^3^, ^4^, ^5^ FI has been highlighted as a serious political and public health concern.^6^ The number of people seeking support from charitable emergency feeding centres, sometimes referred to as food banks, has risen to unprecedented levels, and those with chronic illness are amongst the highest users of food banks in the UK.^7^ The Scottish Health Survey 2017 reported that 18% of individuals living with a chronic illness were also food insecure.^8^ This association between FI and poor health is mediated by compensatory eating behaviours and limited dietary options, as a result of food costs and food bank supplies.^2^ FI not only hinders people's ability to manage their condition, but the associated stress and uncertainty can lead to poor coping behaviours that impact on self‐management.^9^

FI is associated with an increased risk of developing Type 2 diabetes and poorer glycaemic control.^10^, ^11^ Diabetes self‐management requires constant access to high‐quality foods to optimise glycaemic control.^12^ However, healthcare professionals (HCPs) in the UK are encountering patients who struggle to adhere to recommended diets due to financial challenges and associated stress.^3^, ^13^ While the extent of this issue is unclear, managing diabetes in the context of financial hardship and FI poses significant challenges, complicating care and control.

National Institute for Health and Care Excellence (NICE) guidelines for HCPs working in diabetes care concentrate on encouraging a balanced diet and screening patients for malnutrition.^12^ However, there is currently no specific UK guidance recommending HCPs ask patients about potential barriers to a healthy diet. There are strong practical and ethical motives for having conversations around FI in consultations,^2^ particularly in the context of the current cost of living crisis. Previous research has indicated that HCPs experience practical and ethical uncertainty about how to identify and respond to FI among their patients.^9^ There is also concern about the efficacy of food banks in addressing FI, particularly for people living with health conditions.^14^, ^15^, ^16^, ^17^

It is important to examine HCPs' perspectives and approaches to supporting individuals living with diabetes and FI. This study focuses on the perspectives and approaches of HCPs working in a Scottish Health Board covering urban and rural populations. The study objectives were to identify:

1. What factors influence HCPs raising the issue of FI in their consultations with patients with diabetes.
2. What adjustments HCPs make, if any, to their diabetes self‐management support in the context of FI.
3. Whether HCPs feel they have adequate knowledge and training to (i) make appropriate adjustments to care for patients with diabetes and FI and (ii) signpost them to appropriate resources.

## 2 RESEARCH DESIGN AND METHODS

### 2.1 Participants

Participants were recruited by email invitation sent to the Health Board's Diabetes Managed Clinical Network and the diabetes secondary care email list in June 2022. Participants met the following inclusion criteria: (i) HCP providing direct self‐management support to patients with Type 1 or Type 2 diabetes, (ii) working in primary or secondary care. This study received sponsorship and ethical approval from the local university Ethics Review Board (ID: 2331), and approval from the Health Board area's NHS Research and Development division.

### 2.2 Design and procedure

Data were collected through recorded, in‐depth semi‐structured interviews lasting 25–50 min, between July and September 2022. In light of COVID‐19 guidelines for social distancing, remote data collection methods were used. Participants provided informed consent before participating in an interview via Microsoft Teams. An interview topic guide was used to guide the interviews and support data collection, and qualitative field notes were collated to document initial observations, reflections and contextual information from interviews. The topic guide and its use in context were reviewed after the first three interviews to ensure that it was generating the anticipated data needed to meet the study objectives. No modifications were made following this review as it was deemed fit for purpose.

The interview questions were informed by the Capability, Opportunity, Motivation and Behaviour (COM‐B) model of behaviour change.^18^ This model proposes that the performance of a behaviour is dependent on an individual's capability, opportunity and motivation. There were multiple behaviours of interest in this study, including HCPs raising the issue of FI, adjusting care following FI‐related information, and signposting to appropriate resources. In this model, capability refers to an individual's physical and psychological ability to perform the behaviour. Opportunity refers to external factors that make performance of the behaviour possible, including physical and social opportunities bestowed by the environment, such as time, resources, social cues, and cultural norms. Motivation refers to both automatic and reflective processes that influence the behaviour, including beliefs, intentions, evaluations, impulses, and emotions.

### 2.3 Data analysis

A framework method was used to identify, describe and interpret common and exceptional features of data.^19^, ^20^ A combined deductive and inductive approach was applied to address the objectives, whilst allowing for the discovery of unexpected features and themes. Initially, each transcript was read repeatedly by SM and AA until a sound preliminary understanding was achieved. The analysis involved initial coding, identifying a thematic framework, applying the framework to data, charting to summarise data, and finally, identifying and interpreting emergent themes and subthemes related to the COM‐B model.^18^ The coding of data and interpretation of themes and subthemes were reviewed by 3 researchers (SM, AA, FD), and any areas of difference were discussed until consensus was reached. Final themes and subthemes were supported by illustrative quotes (Tables 1, 2, 3).

**TABLE 1:** Key themes and subthemes for raising the issue.

Key theme	Subtheme	Illustrative quote
1. Practitioner level determinants	Personal feelings about raising this issue	*“I feel quite comfortable and relaxed about doing that because… we've seen that it can completely change the nature of the consultation you're having with someone.”* [*P8*]
	Perceived lack of solution or resources to offer patients	*“I don't feel I'm offering any genuine solutions to people, you know, and I'm aware of that.”* [*P7*]
	Knowledge/skills in raising the issue or offering support	*“…it's quite a tricky subject to discuss, but I'm happy enough to discuss it. It would just be how helpful I might be. I might not be all that helpful. That's the thing.”* [*P3*]
	Concerns about patient's response to issue being raised	*“I think I'd have to make sure that I wasn't, umm, making people feel uncomfortable.”* [*P10*]
	Assumption that some HCPs have more opportunity to raise the issue than others	*“I suppose it's this maybe more on us on psychology. You have the time to explore these, you know, issues in a bit more depth. I suppose, Dietitians, because that's all about food and eating, you know, probably more so.”* [*P2*]
	Awareness of food insecurity as an issue	*““I don't think it's something I've ever explicitly asked…perhaps it's naive of me to make an assumption that everyone can feed themselves.”* [*P6*]
2. Consultation and/or organisational factors	Time availability	*“Probably it's time constraint…It's knowing that at the moment I have a 20 minute slot for a return patient*. [*P8*]
	Consultation delivery method and setting	*“…we're going to have to rebuild post pandemic when much of what we've been doing has been telephone and even VC orientated. I think we'll lose some of the subtlety of what happens in a consultation when it's face to face.”* [*P8*]
	Frequency of patient contact	*“Because they only see me for what? Maybe the maximum, maybe half an hour, once a year? So you know, there's a limit to what I can do and, you know, limit then to even, even if I make suggestions and even if I point them towards other things. I really have no way of following up how, how, what their progress is until I see them again.”* [*P3*]
	Prioritisation of food insecurity issues	*“…when people are coming in for their review appointments, there's kind of a specific list of things they need to check and run through, review results, look at medications, and actually that opportunity to sit down and have that conversation in a very short space of time could be very difficult.”* [*P2*]
	Lack of routine food insecurity screening	*“…if we made it part of like normal conversation, normal consultations, people wouldn't feel it's awkward or it wouldn't be as unexpected if it was just part of routine kind of screening.”* [*P2*]
3. Perceptions of patient willingness to disclose FI	Willingness to disclose food insecurity	*“…some people are so proud, aren't they? And they don't want to say and, but others are quite, you know, when you begin to approach the subject, they're quite happy then to say, well, actually yes it is an issue.”* [*P13*]
	Patient‐practitioner relationship	*“…it's mostly around relationship building to try and coax that information out of them.”* [*P4*]
	Fear of being judged by HCPs	*“But they also I think do fear criticism of what they're eating. It's a very touchy subject sometimes with some patients.”* [*P3*]
	Expectation of being told what to do by HCPs	*“It's a tricky one because there is that perception that we are telling them what to do rather than understanding just how difficult it really is.”* [*P3*]
	Appearance and presentation in consultations	*“…people can come very well done up to clinic and you know…that's not what actually they're normally representing like, you know, that's not their reality.”* [*P4*]
	Engagement with health services	*“…unless you come to a clinic appointment or they engage with us, we wouldn't know that there was any kind of access issues.”* [*P15*]
	Beliefs about what to share with HCPs	*“I think there is a barrier kind of to what patients feel that they should be discussing compared to perhaps what they should just say.”* [*P12*]
	Perceived stigma around financial strain and/or food insecurity	*“There was a big stigma, though, about putting your hand up and saying, actually, I really can't cook.”* [*P8*]
4.A multi‐disciplinary approach	Sharing information with or referring to other HCPs	*“…if I feel it's beneficial for them, I will encourage them to speak with a dietician in more detail about it, because I know that they can get them a lot more information and, and a lot more advice.”* [*P3*]
	Asking other HCPs for support with food insecurity‐related conversations	*“…we're constantly going to the psychologist linked with our team to say how do we get this person to open up?”* [*P4*]

**TABLE 2:** Key themes and Subthemes for adjustments to self‐management support.

Key theme	Subtheme	Illustrative quote
1. Tailoring practice	Recommendations, advice and/or goals	*“I mean frequently we're adjusting our recommendations round what somebody is able to do or willing to do.”* [*P1*]
	Diabetes treatment strategies	*“…we've changed their insulin strategies so that they are more flexible in what they choose to eat, rather than being forced to keep up with a prescriptive strategy that we would require them to eat consistently and regularly every day to avoid hypos.”* [*P8*]
	No adjustments to practice	*“…the ability to access food has never, has never really been a topic of conversation in it's in its own right. So no, not really.”* [*P9*]
2. Practical support	Signposting to or accessing resources	*“I've had to access the food bank on patient's behalf.”* [*P13*]
	Improving patient access to treatments	*“We have made sure that folks have access to, umm, probably enhanced quantities of hypo treatments because we know that…For some people, this is really beginning to stretch their budgets, so we're, we're mindful to make sure that there's perhaps a more generous supply on prescription.”* [*P8*]
	Introducing strategies to help with food insecurity‐related stress	*“…if we can at least introduce some strategies to, to help them cope more effectively with stress, that's gonna be helpful for their diabetes management.”* [*P2*]
	Discussing patient's financial situation and possible changes to spending	*“So it's, you know, can you use your skills of trying to encourage them to reduce maybe them wanting to do that and spending the money on that and divert the money elsewhere.”* [*P13*]
3. Patient‐practitioner rapport	Acknowledging and/or normalising patient challenges	*“…it's just finding other ways to improve their self‐management that's not so focused on eating and just hopefully normalizing that a little more is, you know, as much as possible that there are some things that we can do to, to, in terms of self‐management to improve diabetes.”* [*P2*]
	Addressing patient's beliefs and knowledge of self‐management	*“Oh it's about patient understanding as well. I think sometimes it's you know they've maybe had their own beliefs or misunderstanding and, and it's just clarifying well actually, you know, you don't have to spend lots of money on it, you can eat like that.”* [*P13*]
	Building rapport and a positive patient‐practitioner relationship	*“We're not hands on people we're, we're talking people, we don't do any hands on stuff. So we are very much conversation and trying to motivate and encourage, and encourage self‐management.”* [*P15*]

**TABLE 3:** Key themes and subthemes for HCPs' perceptions of their knowledge and capability related to food insecurity and diabetes care.

Key theme	Subtheme	Illustrative quote
1. Perceived knowledge and capability for tailoring care	Awareness of professional knowledge, expertise and/or capabilities	*“I feel that I know enough. Umm, about the physiological aspects of diet and diabetes.”* [*P3*]
	Confidence in adjusting care	*“I feel probably fairly confident. It's probably part of the work we would already do anyways”* [*P2*]
	Concern or distress regarding professional limitations	*“…that's a worry for me, to have to then go home and still bear all that as well, to think there's people out there that I haven't been able to help fully.”* [*P13*]
2. Knowledge of available resources and process for signposting	Extent of knowledge	*“I don't have any knowledge of the benefits system or what, what, what things are out there for different categories of people.”* [*P9*]
	Limited knowledge of resources outside of NHS	*“I think we're still quite silo working within NHS, I think we work as an NHS service. We're not really fully aware of what's happening within local areas.”* [*P15*]
	Limited knowledge of resources in rural areas	*“I could probably tell you more so what's available in the city. But if it came to Shire and Moray, that would be me just Googling away.”* [*P2*]
	Challenges in keeping up‐to‐date with resource availability	*“Keeping up to date is hugely difficult when you're trying to keep up with all that you have to keep up in your own profession.”* [*P13*]
	Negative emotions stemming from perceived lack of knowledge	*“I'm often a bit feel a bit powerless to be able to help them solve it because I don't know where the nearest food bank is.”* [*P1*]
	Limited knowledge of how to access and signpost resources	*“I wouldn't know how to access food banks…I wouldn't know necessarily how to advise someone if I thought that was something that, that they might find useful.”* [*P3*]
3. Efforts to overcome professional limitations	Increasing knowledge of available resources	*“I know about some food banks or some organizations that I could like direct people to, but I wouldn't really know an awful lot about it. And that would be something that I probably have to go away after a session or, you know, before a session and have a think about and look for.”* [*P2*]
	Reliance on patient knowledge of available resources	*“I mean often I would just throw it out there and just say well, do you know of any food banks in your local area?”* [*P1*]
	Involvement of other HCPs	*“…knowing what the practice team can offer as well can be helpful…they often have a much better knowledge locally as to what's available.”* [*P1*]
4. Consideration of wider socioeconomic issues	Perceived impact of adjusting care	*“Even if we can give out that information as on NHS kind of service, how do we then support the families to actually manage that with the kind of, umm, financial things that are going on just now.”* [*P4*]

## 3 RESULTS

### 3.1 Participant characteristics

Fifteen participants were recruited: Specialty Doctors (*n* = 2), Consultant Diabetologists (*n* = 4), Health Psychologists (*n* = 3), Clinical Psychologist (*n* = 1), Clinical Associate in Applied Psychology (n = 1), Diabetes Dieticians (*n* = 3), Paediatric Diabetes Specialist Nurse (*n* = 1). The majority worked in secondary care (*n* = 12), with one participant working in both primary and secondary care settings, and the remaining two participants in primary care.

### 3.2 Key themes and subthemes

The findings revealed themes related to HCPs raising the issue of FI, making adjustments to self‐management support, and their perceptions of their knowledge and capability related to FI and diabetes care.

### 3.3 Raising the issue

#### 3.3.1 Practitioner level determinants

It was common for participants to reflect on their personal feelings, knowledge and skills in raising the issue of FI, or being able to offer appropriate support to patients. Some participants reported feeling comfortable and relaxed about raising the issue, while others felt it was difficult or awkward. One participant believed that their own limited understanding and awareness of FI had been a barrier in consultations. Another participant shared that they would feel more comfortable asking about FI if they could signpost patients to an appropriate service for support. A perceived lack of solutions or resources was a common factor that inhibited bringing up of this issue:

“I would rarely open up a wound that I cannot close…if you expose something like that in consultation, then we need to have something to help them.” [P7]

Others raised concerns about how patients may respond to being asked about FI, describing worries about increasing patients' stress, making them feel uncomfortable or offending them. Some believed that certain professionals, particularly dieticians and psychologists, have more responsibility or opportunities to raise issues of FI in consultations. This was recognised by several of the dieticians and psychologists themselves.

#### 3.3.2 Consultation and/or organisational factors

Participants commonly talked about the need for adequate time in consultations to address FI. Many participants reported limited time allotted for each consultation, particularly with regard to return appointments. Another common factor was the need for prioritisation of other clinical issues within consultations, with participants highlighting the impact of competing work demands and pressures such as reviewing results and looking at medications, as illustrated:

“…there's so many other things that they're having to do at the moment and so many other topics they're having to talk about. I'm not sure that they would prioritise food insecurity versus hypo treatment or sick day rules.” [P11]

Participants talked about additional enablers and barriers, from the frequency of contact with patients to the consultation method and setting:

“I think sometimes the clinic rooms, if they're very clinical, the patients become very clinical and just deal with the clinical situation rather than being able to relax.” [P15]

A lack of routine screening of FI within normal practice was also identified as a barrier to raising the issue, with one participant stating that they would find it less awkward to ask about FI if it was a normal part of routine screening.

#### 3.3.3 Perceptions of patient willingness to disclose FI

It was common for participants to talk about patients' willingness to disclose experiences of FI as a potential enabler or barrier to these discussions, highlighting a need for good patient‐practitioner relationships. Many participants believed patients may be reluctant to discuss issues of FI with HCPs, which they associated with patients' fears of judgement or criticism, their beliefs about what information they should share with HCPs, and feelings of embarrassment or shame. It was also highlighted that a patient's appearance and presentation when attending a consultation could be an enabler or barrier, as patients may make efforts to hide their personal circumstances:

“…people can come very well done up to clinic and…that's not what actually they're normally representing like, you know that's not their reality.” [P4]

One participant believed that there had been a decline in the societal stigma associated with issues of FI or financial strain since the COVID‐19 pandemic and felt it was easier to ask questions about these issues as a result. Another participant believed that since the pandemic, people have generally been more open about the challenges they have faced.

#### 3.3.4 A multi‐disciplinary approach

Approximately half of participants highlighted the use of a multi‐disciplinary approach to help address FI, by sharing information with other HCPs or referring patients on for specialised support. However, one participant highlighted practical challenges to this:

“…it's a very large area and you've got … different health and social care partnerships and you know as much as we try and like link up, it's very difficult to keep track of everything that's going on.” [P2]

### 3.4 Adjustments to self‐management support

#### 3.4.1 Tailoring practice

When asked about supporting patients living in the context of FI, there were common accounts of HCPs adjusting recommendations that were associated with tailoring advice and agreeing goals that were realistic in support of gradual behaviour change. Several participants described working with patients to adjust their treatment strategies:

“…we've changed their insulin strategies so that they are more flexible in what they choose to eat, rather than being forced to keep up with a prescriptive strategy that we would require them to eat consistently and regularly every day.” [P8]

One participant shared that they had not made any adjustments to their practice and felt unsure what they could do to support a patient living with FI.

#### 3.4.2 Practical support

Most participants reported that they had or would try to provide patients with practical support, including signposting patients to relevant resources, such as food banks or financial aid. One participant described providing more direct support by accessing those resources on the patient's behalf:

“I spent an hour phoning food banks to try and get a guy out of hospital because he had no food at home and we couldn't discharge him.” [P1]

Others had helped patients to apply for financial support or improved their access to treatments. Less common practices included strategies to support patients with experiences of stress, and discussing a patient's financial situation to explore possible changes in spending.

#### 3.4.3 Patient‐practitioner rapport

Building rapport and a positive patient‐practitioner relationship were thought to be an important aspect of providing effective self‐management support. Participants described acknowledging and normalising patients' difficulties, building trust, providing reassurance, and using patient‐centred discussions to bolster motivation and encourage behaviour change. One participant highlighted that this may involve addressing patients' beliefs and possible misunderstandings about healthy eating and associated financial costs.

### 3.5 HCPs' perceptions of their knowledge and capability related to food insecurity and diabetes care

#### 3.5.1 Perceived knowledge and capability for tailoring care

Most participants felt they had adequate knowledge or expertise to provide diabetes self‐management support for those living with FI. Others shared that they had limited understanding of how to address FI or have constructive conversations about related issues. One participant expressed concern that they should do more to address FI, while another cited feelings of distress related to their perceived inability to provide fundamental solutions for those living with these difficulties:

“…that's a worry for me, to have to then go home and still bear all that as well, to think there's people out there that I haven't been able to help fully.” [P13]

#### 3.5.2 Knowledge of available resources and process for signposting

Only one participant believed they had a good awareness of available resources to support individuals living with FI. While several participants reported not being aware of any resources for signposting:

“I don't have any knowledge of the benefits system or what, what, what things are out there for different categories of people.” [P9]

One participant shared that their knowledge was particularly limited for resources in rural areas, while another reported limited awareness of resources outside of the NHS, highlighting practical challenges for HCPs in keeping up‐to‐date. Another participant described feeling powerless and frustrated by their perceived limited knowledge of how to access and signpost resources:

“I wouldn't know how to access food banks…I wouldn't know what the process is for someone, so I wouldn't know necessarily know how to advise someone.” [P3]

#### 3.5.3 Efforts to overcome professional limitations

It was common for participants to report speaking or referring patients to other HCPs, such as nurses and dieticians, in an effort to overcome their perceived professional limitations in providing tailored support or signposting to resources. One participant described searching for available resources outside of consultations as a way to increase their knowledge, while another had relied on their patients' own knowledge of available resources when discussing options for support.

#### 3.5.4 Consideration of wider socioeconomic issues

One participant raised uncertainties about the ability of HCPs, and the NHS more generally, to support those living with FI within the current socioeconomic context:

“Even if we can give out that information as an NHS kind of service, how do we then support the families to actually manage that with the…financial things that are going on just now.” [P4]

### 3.6 COM‐B model

The following section brings together our findings in consideration of the COM‐B model. Table 4 presents key themes and subthemes mapped onto the three components; capability, opportunity and motivation.

**TABLE 4:** Summary of themes and subthemes for each COM‐B Domain.

COM‐B Domain	Theme	Sub‐theme
Capability	Practitioner factors	Awareness of professional knowledge, expertise and/or capabilities
		Knowledge/skills in raising the issue or offering support
		Awareness of food insecurity as an issue
	Knowledge of available resources and process for signposting	Extent of knowledge
		Limited knowledge of resources outside of NHS
		Limited knowledge of resources in rural areas
		Challenges in keeping up‐to‐date with resource availability
		Negative emotions stemming from perceived lack of knowledge
		Limited knowledge of how to access and signpost resources
	Efforts to overcome professional limitations	Increasing knowledge of available resources
		Reliance on patient knowledge of available resources
		Involvement of other HCPs
	A multi‐disciplinary approach	Sharing information with or referring to other HCPs
		Asking other HCPs for support with food insecurity‐related conversations
		Assumption that some HCPs have more opportunity to raise the issue than others
	Providing practical support	Signposting to or accessing resources
		Improving patient access to treatments
		Introducing strategies to help with food insecurity‐related stress
		Discussing patient's financial situation and possible changes to spending
	Tailoring practice based on patient circumstances	Recommendations, advice and/or goals
		Diabetes treatment strategies
		No adjustments to practice
Opportunity	Consultation factors	Time availability
		Consultation delivery method and setting
		Frequency of patient contact
		Prioritisation of food insecurity issues
		Lack of routine food insecurity screening
	Patient factors	Willingness to disclose food insecurity
		Patient‐practitioner relationship
		Fear of being judged by HCPs
		Expectation of being told what to do by HCPs
		Appearance and presentation in consultations
		Engagement with health services
		Beliefs about what to share with HCPs
	Patient‐practitioner rapport	Acknowledging and/or normalising patient challenges
		Addressing patient's beliefs and knowledge of self‐management
		Building rapport and a positive patient‐practitioner relationship
	Societal factors	Perceived stigma around financial strain and/or food insecurity
Motivation	Practitioner factors	Personal feelings about raising the issue
		Confidence in adjusting care
		Perceived lack of solution or resources to offer patients
		Concerns about patient's response to issue being raised
		Concern or distress regarding professional limitations
	Consideration of wider social and economic issues	Perceived impact of adjusting care

#### 3.6.1 Capability

Most HCPs reported confidence and expertise in their ability to appropriately adjust guidance and treatment plans for individuals with FI. However, in some cases, professional limitations and personal lack of awareness were recognised as a barrier to HCPs discussing and supporting patients with these issues. Furthermore, only a few HCPs considered how they would support patients with feelings of stress or uncertainty associated with FI.

It was apparent that HCPs had limited awareness of resources available to support patients. Although food banks were commonly identified, HCPs reported limited awareness of the availability and operation of local food banks, and how to support patients in accessing them. Only one participant shared concerns about the suitability of food and long‐term effectiveness of food banks in addressing FI for individuals with diabetes.

#### 3.6.2 Opportunity

HCPs identified a range of external barriers to discussing issues of FI, including the duration, regularity and setting of their consultations, increased work demands, and the absence of a screening tool for FI. The development and maintenance of a good patient‐practitioner relationship and patients’ willingness to disclose FI issues were highlighted as key facilitators. Multidisciplinary working was another commonality across interviews, with HCPs highlighting sharing information and referring patients on to other services for specialised support. Dieticians and psychologists were perceived to have more relevant expertise and increased opportunities to address issues of FI within their practice.

#### 3.6.3 Motivation

HCPs expressed a continuum of feelings about raising the issue of FI, with many perceiving it as a difficult, sensitive or awkward topic of conversation. They also outlined concerns about how patients might respond to being asked about FI. Despite this, the majority of participants shared that they would tailor care and provide practical support or signpost to relevant resources. Not all participants had prior experience of adjusting their practice in this way and some HCPs displayed resistance to raising the issue of FI if they felt they have no practical solutions to offer. Others described feeling burdened, powerless and distressed by their perceived inability to resolve issues of FI.

### 3.7 Ideas for practical solutions

Participants were asked what would help them to better support individuals living with FI and how discussions between patient and practitioner could be improved. Specific FI‐focused training for diabetes care was the most dominant theme. It was thought that this would remind HCPs of the issue during routine consultations and help improve their knowledge and skills in supporting patients. Receiving training that would improve knowledge about local resources and related referral processes were highlighted.

Participants suggested making information more accessible for patients through leaflets or a website to signpost local resources. Some thought having a comprehensive, up‐to‐date list of food banks within their geographic Health Board area, with information about how they operated, would be beneficial. It was commonly thought this type of resource may require a dedicated local expert person or team:

“Maybe we need some sort of dedicated individual in the clinic who might, you know, be the go to person for support and advice about that.” [P3]

One participant advocated for a community hub area where patients could go to discuss their hardships and acquire information about available resources. Others mentioned linking up with other clinics and care settings to understand what they typically offer patients.

Participants highlighted the potential benefits of including questions around FI in a pre‐consultation checklist, as a cue to discuss concerns. Several participants referred to the Making Every Opportunity Count (MEOC) approach to consultations, which had previously been rolled out locally to support patients: a pre‐consultation checklist to raise awareness of patients' difficulties. Some participants mentioned the use of a FI screening measure, which they felt would lessen their responsibility to raise the issue and reduce feelings of awkwardness for both patient and practitioner.

Engaging with third sector resources and initiatives to support patients was a common feature. One participant suggested that local food cooperatives could come to the clinic to sell cheap foods. Being able to offer patients opportunities to learn cooking skills was also highlighted.

## 4 DISCUSSION

This study of secondary care HCPs found that staff do not habitually consider or discuss FI issues within their routine clinical practice. This oversight suggests a significant gap in patient‐centred care that needs to be addressed.

HCPs expressed ethical concerns about how to approach the topic of FI with patients, reflecting a broader uncertainty documented in similar research.^13^ This hesitance is further compounded by a perceived lack of practical solutions. Previous research has evidenced scepticism and pessimism about the effectiveness of food banks as a sustainable solution to FI for individuals with chronic health conditions.^15^ Interestingly, concerns about the suitability and long‐term effectiveness of food banks in addressing FI for individuals with diabetes was not a common feature across interviews.

This study has identified some key issues for NHS services and senior decision‐makers, as well as practical steps to support HCPs in their practice. The absence of a screening tool for FI was seen as an impediment to raising the issue of FI, as previously found.^2^ Given the significant pressures and time constraints in patient consultations, introducing a brief self‐report tool for patients to complete before consultations could help staff efficiently address this topic. Other healthcare systems are increasingly using screening tools for FI, such as the 2‐item Hunger Vital Sign^21^ or the 10‐item Accountable Health Communities Health‐Related Social Needs screening tool.^22^ Adopting similar screening tools could enhance patient‐centred care, help tailor interventions, and mitigate health inequalities. However, it is essential to explore the experiences of patients and staff in primary and secondary care using these tools, and pilot their use, prior to wider implementation, to minimise potential feelings of shame, stress or alienation.^23^

Overall, integrating FI considerations into routine clinical practice is vital to help improve patient experience, health outcomes and promote equity in diabetes care. The NHS Long Term Plan highlights the need to address health inequalities,^24^ and ensuring patients have consistent access to nutritious food supports this goal. By recognising the role of FI in diabetes management, HCPs can provide tailored support to underserved communities and ultimately enhance care effectiveness. Nevertheless, this study raises fundamental questions about what the NHS can do to support individuals challenged by the impact of wider socioeconomic issues beyond its control. There was recognition of healthcare being shaped by the government's policy choices and decisions on social spending, underscoring the need for system‐level changes to support NHS action.^25^

### 4.1 Strengths and limitations

A key strength of this study was the application of the COM‐B model of behaviour change to guide data collection and analysis. This model has been widely used to understand the role of cognitive factors, emotional processes and environmental systems on individuals' health behaviours.^26^, ^27^, ^28^ It has been applied to understand barriers and facilitators to the behaviour and practice of health professionals.^29^, ^30^

This study captured the knowledge, experiences and training needs of a small sample of HCPs predominantly working in a large acute hospital setting in Scotland. Although the findings complement existing literature in the area,^2^, ^9^, ^15^ our sample consisted predominantly of diabetes physicians and psychologists, with limited representation of other HCPs, including diabetes specialist nurses and those based in primary care. It was not possible to recruit additional participants from these key groups, due to limited time and access to relevant networks, as well as a lack of engagement from some groups. This may in part be a result of the substantial work demands and pressures faced by HCPs day‐to‐day, leaving limited time and capacity to engage in research activities. This research has also not explored how community‐based health and social care professionals are dealing with issues of FI. There is a need for further research to capture the perspectives and experiences of HCPs based in primary and community settings who may play a key role in raising and addressing issues of FI, as well as their training and support needs for tailoring diabetes care in this context.

In addition, there was no scope in this study to explore the views of patients living with diabetes and FI, and their experiences of receiving health care. There is a paucity of literature in this important area. Increasing our understanding of patients' experiences may help develop innovative programmes and interventions to support people living with FI, and has the potential to influence social policies to address poverty and health inequalities.

Finally, the role of two of the researchers should be considered, as SM (a health psychologist) and AA (a clinician in the diabetes service) may have influenced responses. It is possible that their clinical experiences and beliefs influenced data interpretation. However, a third researcher, FD (a public health scientist), provided critical reflections and challenged the other researchers' positions and experiences in the final interpretation of the data.

## 5 CONCLUSION

This study highlights to NHS services and senior decision‐makers that FI is not currently core to routine clinical practice in diabetes. The findings suggest that training for staff, accessible information for patients and practitioners, self‐report pro forma, and NHS‐third sector links and pathways may help support HCPs and their patients consider the ways in which FI is impacting self‐management. There is also a need for further research with affected patients and the NHS workforce more widely to gain a better understanding of how to provide high‐quality, effective care within NHS settings.

## CONFLICT OF INTEREST STATEMENT

The authors have no competing interests to report.

## References

[1] Dowler EA , O'Connor D . Rights‐based approaches to addressing food poverty and food insecurity in Ireland and UK. Soc Sci Med. 2012;74(1):44‐51. 22000764 10.1016/j.socscimed.2011.08.036

[2] Knight JK , Fritz Z . Doctors have an ethical obligation to ask patients about food insecurity: what is stopping us? J Med Ethics. 2002;48(10):707‐711. 10.1136/medethics-2021-107409 PMC9554025 34261802

[3] MacLeod MA , Curl A , Kearns A . Understanding the prevalence and drivers of food bank use: evidence from deprived communities in Glasgow. Soc Policy Soc. 2019;18(1):67‐86.

[4] Sosenko F , Livingstone N , Fitzpatrick S . Overview of Food Aid Provision in Scotland. Edinburgh; 2013.

[5] Vozoris NT , Tarasuk VS . Household food insufficiency is associated with poorer health. J Nutr. 2003;133(1):120‐126. 12514278 10.1093/jn/133.1.120

[6] Scottish Government . A healthier Future: Scotland's Diet and Healthy Weight Delivery Plan. Edinburgh; 2018.

[7] Loopstra R , Lalor D . Financial insecurity, food insecurity, and disability: the profile of people receiving emergency food assistance from the Trussell trust foodbank network in Britain. London: The Trussell Trust; 2017.

[8] Scottish Government . The Scottish Health Survey 2017. Edinburgh; 2017.

[9] Douglas F , MacIver E , Yuill C . A qualitative investigation of lived experiences of long‐term health condition management with people who are food insecure. BMC Public Health. 2020;20(1):1‐15. 32859179 10.1186/s12889-020-09299-9 PMC7456079

[10] Gucciardi E , Vahabi M , Norris N , Del Monte JP , Farnum C . The intersection between food insecurity and diabetes: a review. Curr Nutr Rep. 2014;3(4):324‐332. 25383254 10.1007/s13668-014-0104-4 PMC4218969

[11] Seligman HK , Bindman AB , Vittinghoff E , Kanaya AM , Kushel MB . Food insecurity is associated with diabetes mellitus: results from the National Health Examination and nutrition examination survey (NHANES) 1999–2002. J Gen Intern Med. 2007;22(7):1018‐1023. 17436030 10.1007/s11606-007-0192-6 PMC2583797

[12] National Institute for Health and Clinical Excellence . Type 2 Diabetes in Adults: Management. NICE; 2015. 26741015

[13] Douglas F , Machray K , Entwistle V . Health professionals' experiences and perspectives on food insecurity and long‐term conditions: a qualitative investigation. Health Soc Care Community. 2020;28(2):404‐413. 31595585 10.1111/hsc.12872 PMC7027877

[14] Bazerghi C , McKay FH , Dunn M . The role of food banks in addressing food insecurity: a systematic review. J Community Health. 2016;41:732‐740. 26728281 10.1007/s10900-015-0147-5

[15] Douglas F , MacKenzie F , Ejebu OZ , et al. “A lot of people are struggling privately. They don't know where to go or they're not sure of what to do”: frontline service provider perspectives of the nature of household food insecurity in Scotland. Int J Environ Res Public Health. 2018;15(12):2738. 30518162 10.3390/ijerph15122738 PMC6313537

[16] Mossenson S , Giglia R , Pulker CE , Chester M , Pollard CM . Dietary risk of donated food at an Australian food bank: an audit protocol. BMC Nutr. 2023;9(1):1‐12. 37277849 10.1186/s40795-023-00719-8 PMC10243076

[17] Oldroyd L , Eskandari F , Pratt C , Lake AA . The nutritional quality of food parcels provided by food banks and the effectiveness of food banks at reducing food insecurity in developed countries: a mixed‐method systematic review. J Hum Nutr Diet. 2022;35(6):1202‐1229. 35112742 10.1111/jhn.12994 PMC9790279

[18] Michie S , Van Stralen MM , West R . The behaviour change wheel: a new method for characterising and designing behaviour change interventions. Implement Sci. 2011;6(1):1‐12. 21513547 10.1186/1748-5908-6-42 PMC3096582

[19] Goldsmith LJ . Using framework analysis in applied qualitative research. Qual Rep. 2021;26(6):2061‐2076.

[20] Ritchie J , Lewis J , Nicholls CM , Ormston R , eds. Qualitative Research Practice: a Guide for Social Science Students and Researchers. Sage; 2013.

[21] Hager ER , Quigg AM , Black MM , et al. Development and validity of a 2‐item screen to identify families at risk for food insecurity. Pediatrics. 2010;126(1):e26‐e32. 20595453 10.1542/peds.2009-3146

[22] Billioux A , Verlander K , Anthony S , Alley D . Standardized screening for health‐related social needs in clinical settings: the accountable health communities screening tool. NAM Perspect. 2017;7(5):1‐9. doi:10.31478/201705B Accessed 30 May, 2024.

[23] Easton P , Entwistle VA , Williams B . How the stigma of low literacy can impair patient‐professional spoken interactions and affect health: insights from a qualitative investigation. BMC Health Serv Res. 2013;13(1):319. 23958036 10.1186/1472-6963-13-319 PMC3751726

[24] England NHS . NHS Long Term Plan. 2019 Retrieved from https://www.longtermplan.nhs.uk/

[25] Buzelli L , Dunn P , Scott S , Gottlieb L , Alderwick H . A framework for NHS action on social determinants of health. The Health Foundation. 2022 Accessed 30 May, 2024. https://www.health.org.uk/publications/long‐reads/a‐framework‐for‐nhs‐action‐on‐social‐determinants‐of‐health

[26] Jackson C , Eliasson ÂL , Barber N , Weinman J . Applying COM‐B to medication adherence: a suggested framework for research and interventions. Eur Health Psychol. 2014;16(1):7‐17.

[27] Ojo SO , Bailey DP , Hewson DJ , Chater AM . Perceived barriers and facilitators to breaking up sitting time among desk‐based office workers: a qualitative investigation using the TDF and COM‐B. Int J Environ Res Public Health. 2019;6(16):2903. 10.3390/ijerph16162903 PMC6720704 31416112

[28] Szinay D , Perski O , Jones A , Chadborn T , Brown J , Naughton F . Perceptions of factors influencing engagement with health and wellbeing apps: a qualitative study using the COM‐B model and theoretical domains framework as an analytical framework. JMIR Mhealth Uhealth. 2021;9(12):e29098. 34927597 10.2196/29098 PMC8726027

[29] Alexander KE , Brijnath B , Mazza D . Barriers and enablers to delivery of the healthy kids check: an analysis informed by the theoretical domains framework and COM‐B model. Implement Sci. 2014;9(1):1‐14. 24886520 10.1186/1748-5908-9-60 PMC4047437

[30] Mazza D , Chapman A , Michie S . Barriers to the implementation of preconception care guidelines as perceived by general practitioners: a qualitative study. BMC Health Serv Res. 2013;13(1):1‐8. 23368720 10.1186/1472-6963-13-36 PMC3565953

